# Serum syndecan-1 reflects organ dysfunction in critically ill patients

**DOI:** 10.1038/s41598-021-88303-7

**Published:** 2021-04-23

**Authors:** Keiko Suzuki, Hideshi Okada, Kazuyuki Sumi, Hiroyuki Tomita, Ryo Kobayashi, Takuma Ishihara, Yoshinori Kakino, Kodai Suzuki, Naomasa Yoshiyama, Ryu Yasuda, Yuichiro Kitagawa, Tetsuya Fukuta, Takahito Miyake, Haruka Okamoto, Tomoaki Doi, Takahiro Yoshida, Shozo Yoshida, Shinji Ogura, Akio Suzuki

**Affiliations:** 1 Department of Pharmacy, Gifu University Hospital, 1-1 Yanagido, Gifu, 501-1194 Japan; 2 Department of Emergency and Disaster Medicine, Gifu University Graduate School of Medicine, 1-1 Yanagido, Gifu, 501-1194 Japan; 3 Department of Tumor Pathology, Gifu University Graduate School of Medicine, Gifu, Japan; 4 Innovative and Clinical Research Promotion Center, Gifu University Hospital, Gifu, Japan

**Keywords:** Biomarkers, Statistics, Predictive markers

## Abstract

Syndecan-1 (SDC-1) is found in the endothelial glycocalyx and shed into the blood during systemic inflammatory conditions. We investigated organ dysfunction associated with changing serum SDC-1 levels for early detection of organ dysfunction in critically ill patients. To evaluate the effect of SDC-1 on laboratory parameters measured the day after SDC-1 measurement with consideration for repeated measures, linear mixed effects models were constructed with each parameter as an outcome variable. A total of 94 patients were enrolled, and 831 samples were obtained. Analysis using mixed effects models for repeated measures with adjustment for age and sex showed that serum SDC-1 levels measured the day before significantly affected several outcomes, including aspartate aminotransferase (AST), alanine transaminase (ALT), creatinine (CRE), blood urea nitrogen (BUN), antithrombin III, fibrin degradation products, and D-dimer. Moreover, serum SDC-1 levels of the prior day significantly modified the effect between time and several outcomes, including AST, ALT, CRE, and BUN. Additionally, increasing serum SDC-1 level was a significant risk factor for mortality. Serum SDC-1 may be a useful biomarker for daily monitoring to detect early signs of kidney, liver and coagulation system dysfunction, and may be an important risk factor for mortality in critically ill patients.

## Introduction

The vascular endothelium, which consists of a thin monolayer of endothelial cells, lines the entire circulatory system from the heart to the smallest capillaries where it is exposed to the circulating blood. All healthy endothelia are coated with the sugar protein glycocalyx, which is composed of membrane-bound proteoglycans, glycoproteins, glycosaminoglycans, and adherent plasma proteins^1–4^. It performs several functions that are necessary for vascular homeostasis, including regulation of vascular permeability and microvascular tone, inhibition of microvascular thrombosis, and regulation of leukocyte adhesion and migration on the endothelium^5–8^.

Systemic inflammatory conditions lead to endothelial dysfunction, which in turn increases paracellular permeability and the outflow of albumin/fluid into the interstitial space^5^. This effect is thought to be caused by glycocalyx disruption. Schmidt et al. demonstrated that degradation of endothelial glycocalyx contributes to the pathogenesis of acute respiratory distress syndrome in the lipopolysaccharide (LPS)-induced experimental sepsis mouse model and humans^9,10^. Similarly, recent reports indicate that severe disruption to capillary endothelial glycocalyx leads to damaged organs, including the heart, kidneys, lungs, liver, and brain, as observed by scanning and transmission electron microscopy, suggesting that endothelial dysfunction occurs in the process of organ damage^11–13^. Therefore, glycocalyx fragments shed into the blood during sepsis may serve as a clinically relevant biomarker of organ dysfunction, given the pathophysiologic implications of glycocalyx degradation^14^.

Serum syndecan-1 (SDC-1), the core protein in heparan sulfate proteoglycan, is found in the endothelial glycocalyx. In clinical research, serum SDC-1 has been used as an endothelial injury marker of several diseases, including chronic kidney disease^15^, diabetes^16,17^, cardiovascular disease^18^, hypertriglyceridemia^19^ and sepsis^20,21^. Moreover, several indicators have demonstrated that serum SDC-1 levels are significantly correlated with the Sequential Organ Failure Assessment **(**SOFA) score in patients with sepsis^22–24^, and that non-survivors have significantly higher SDC-1 levels than survivors with sepsis^21,24,25^. However, it remains unknown which organs show changing serum SDC-1 levels when damaged, and whether SDC-1 is a useful predictive marker for early detection of dysfunction in these organs.

The aim of this study was to investigate the association between organ dysfunction and changing serum SDC-1 levels in critically ill patients and to identify organ dysfunctions that may be amenable to early detection through daily monitoring of SDC-1 levels.

## Results

### Patient characteristics

A total of 314 patients were admitted to the ICU at Gifu University Hospital during the study period. Of these, 220 patients were excluded for the following reasons: under 18 years old (n = 17), discharged from the ICU within 72 h (n = 79), undergoing hemodiafiltration (n = 9), did not give consent to participate in the present study (n = 115). Thus, the remaining 94 patients (70 men and 24 women) were enrolled in this study. A total of 831 samples were obtained, and the median number of measurements per patients was 8 (IQR, 4.25–15). Patient characteristics are shown in Table 1. The median age was 67.0 years (IQR, 53.3–77.0), median SOFA score on ICU admission was 6.0 (IQR, 4.0–9.8), and median duration of ICU stay was 10.5 days (IQR, 6.0–19.0). The most common condition upon admission was trauma (n = 40, 42.6%), followed by myocardial infarction (n = 15, 16.0%), sepsis (n = 10, 10.6%), burn (n = 6, 6.4), heatstroke (n = 3, 3.2%), heart failure (n = 2, 2.1%), acute aortic dissection (n = 2, 2.1%), ventricular fibrillation (n = 2, 2.1%), soft tissue infection (n = 2, 2.1%) and hypoglycemia (n = 2, 2.1%). The most common source of sepsis was *Klebsiella species* (n = 4), followed by *Streptcoccus species* (n = 3), *Methicillin-susceptible Staphylococcus epidermidis* (n = 1), *Escherichia coli* (n = 1) and unknown (n = 1). No patients had sepsis due to severe acute respiratory syndrome coronavirus 2 (SARS-CoV-2).

**Table 1:** Patient demographics.

Age, years, median (IQR)	67 (53.3–77.0)
Sex, male/female, n (%)	70 (74.5)/24 (25.5)
Weight, kg, median (IQR)	60.4 (53.1–75.1)
SOFA score on ICU admission, median (IQR)	6.0 (4.0–9.8)
**Condition, n (%)**	
Trauma	40 (42.6)
Myocardial infarction	15 (16.0)
Sepsis	10 (10.6)
Burn	6 (6.4)
Heatstroke	3 (3.2)
Heart failure	2 (2.1)
Acute aortic dissection	2 (2.1)
Ventricular fibrillation	2 (2.1)
Soft tissue infection	2 (2.1)
Hypoglycemia	2 (2.1)
Other	10 (10.6)
**Source of sepsis, n (%)**	
*Klebsiella species*	4 (4.3)
*Streptcoccus species*	3 (3.2)
*Methicillin-susceptible Staphylococcus epidermidis*	1 (1.0)
*Escherichia coli*	1 (1.0)
Unknown	1 (1.0)
Length of ICU stay, days, median (IQR)	10.5 (6.0–19.0)

All data indicate median, 25–75th percentile unless otherwise indicated.

*ICU* intensive care unit, *IQR* interquartile range.

### Association of serum SDC-1 levels measured the day before with various parameters

The results of the mixed effects models with adjustment for age and sex are shown in Table 2. Serum SDC-1 levels measured the day before laboratory parameter measurements significantly affected AST [coefficient (*β*) = 0.0042, 95% confidence interval (CI) 0.0018–0.0065, *P* = 0.001, *q* = 0.003], ALT (*β* = 0.0034, 95% CI 0.0012–0.0057, *P* = 0.003, *q* = 0.009), CRE (*β* = 0.002, 95% CI 0.0012–0.0028, *P* < 0.001, *q* < 0.001), BUN (*β* = 0.0015, 95% CI 0.0002–0.0028, *P* = 0.019, *q* = 0.044), T-Bil (*β* = − 0.0026, 95% CI − 0.0041 to − 0.0011, *P* = 0.001, *q* = 0.003), fibrin degradation products (FDP; *β* = 0.154, 95% CI 0.023–0. 285, *P* = 0.021, *q* = 0.038), antithrombin III (AT III; *β* = − 0.0015, 95% CI − 0.1307 to − 0.0107, *P* = 0.021, *q* = 0.042) and total volume of infusion per day (coefficient = − 17.8331, 95% CI − 25.2211 to − 10.445, *P* < 0.001, *q* < 0.001). Although not statistically significant, serum SDC-1 levels of the prior day also tended to affect LD (*β* = 0.0012, 95% CI 0–0.0024, *P* = 0.056, *q* = 0.089) and D-dimer (*β* = 0.0821, 95% CI 0.0135–0.1507, *P* = 0.019, *q* = 0.051). Moreover, the interaction term of time and SDC-1 was significant for AST (*P* < 0.001, *q* = 0.002), ALT (*P* < 0.001, *q* = 0.001), LD (*P* < 0.001, *q* < 0.001), ALP (*P* < 0.001, *q* = 0.004), CRE (*P* = 0.009, *q* = 0.021), BUN (*P* = 0.018 *q* = 0.037), T-Bil (*P* = 0.001, *q* = 0.01) and total volume of infusion per day (*P* < 0.001, *q* < 0.001).

**Table 2:** Linear mixed effects models that did and did not consider the interaction of time and SDC-1.

Outcome	No consideration for interaction between time and SDC-1					Interaction between time and SDC-1	
	Coefficient^a^	95% LCL	95% UCL	*P-*value	*q-*value^b^	*P-*value	*q-*value^b^
TP	0.0012	− 0.0037	0.0061	0.638	0.729	0.362	0.482
ALB	0.0015	− 0.0013	0.0042	0.289	0.385	0.111	0.198
log_CK	0.0013	− 0.0017	0.0043	0.401	0.494	0.861	0.861
log_AST	0.0042	0.0018	0.0065	0.001	0.003	< 0.001	0.002
log_ALT	0.0034	0.0012	0.0057	0.003	0.009	< 0.001	0.001
log_LD	0.0012	0	0.0024	0.056	0.089	< 0.001	< 0.001
log_ALP	0.0002	− 0.0010	0.0015	0.695	0.741	0.001	0.004
log_ChE	− 0.0014	− 0.0032	0.0004	0.115	0.168	0.365	0.449
log_CRE	0.0020	0.0012	0.0028	< 0.001	< 0.001	0.009	0.021
log_BUN	0.0015	0.0002	0.0028	0.019	0.044	0.018	0.037
log_TG	0.0032	− 0.0455	0.0519	0.874	0.874	0.805	0.859
log_T-Bil	− 0.0026	− 0.0041	− 0.0011	0.001	0.003	0.004	0.01
FDP	0.1540	0.0230	0.2850	0.021	0.038	0.336	0.489
D-dimer	0.0821	0.0135	0.1507	0.019	0.051	0.152	0.243
AT III	− 0.0707	− 0.1307	− 0.0107	0.021	0.042	0.694	0.793
Volume of infusion per day	− 17.8331	− 25.2211	− 10.4450	< 0.001	< 0.001	< 0.001	< 0.001

All mixed effects models were adjusted for age and sex.

*SDC-1* syndecan-1, *TP* total protein, *ALB* albumin, *CK* creatine kinase, *AST* aspartate aminotransferase, *ALT* alanine aminotransferase, *LD* lactate dehydrogenase, *ALP* alkaline phosphatase, *ChE* cholinesterase, *CRE* creatinine, *BUN* blood urea nitrogen, *TG* triglyceride, *T-Bil* total-bilirubin, *FDP* fibrin degradation product, *AT III* antithrombin III, *LCL* lower confidence limit, *UCL* upper confidence limit.

^a^Coefficient indicates the increase in the mean value of an outcome when SDC-1 increases by 10.

^b^The *q*-value was calculated using the Benjamini–Hochberg method.

### Predicted parameter values over time by serum SDC-1 level

**Figure 1:**
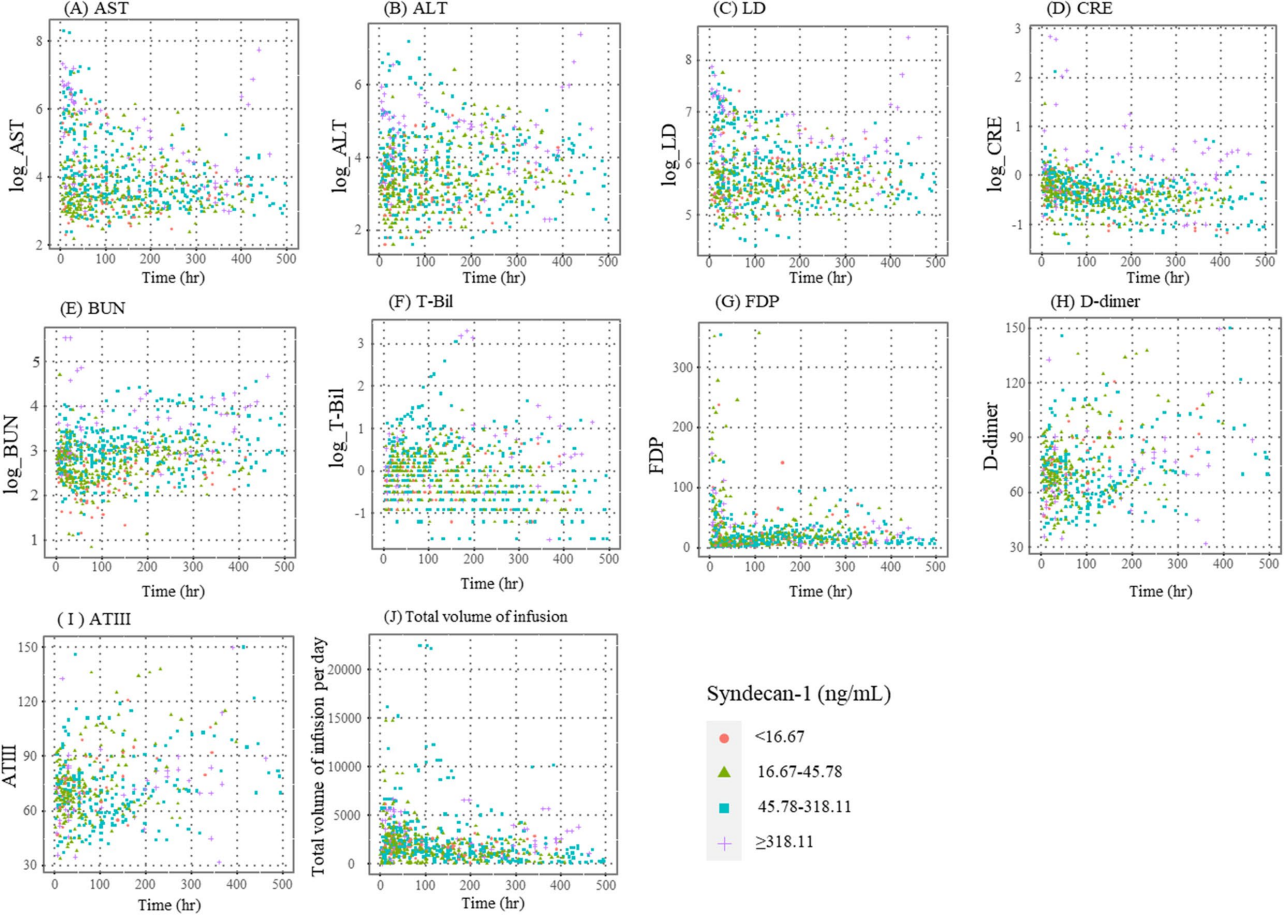
Scatter plots of levels of each parameter over time in patients with serum SDC-1 levels of the prior day of < 16.67 ng/mL (red circle), 16.67–45.78 ng/mL (green triangle), 45.78–318.11 ng/mL (blue square) and < 318.11 ng/mL (purple cross).

**Figure 2:**
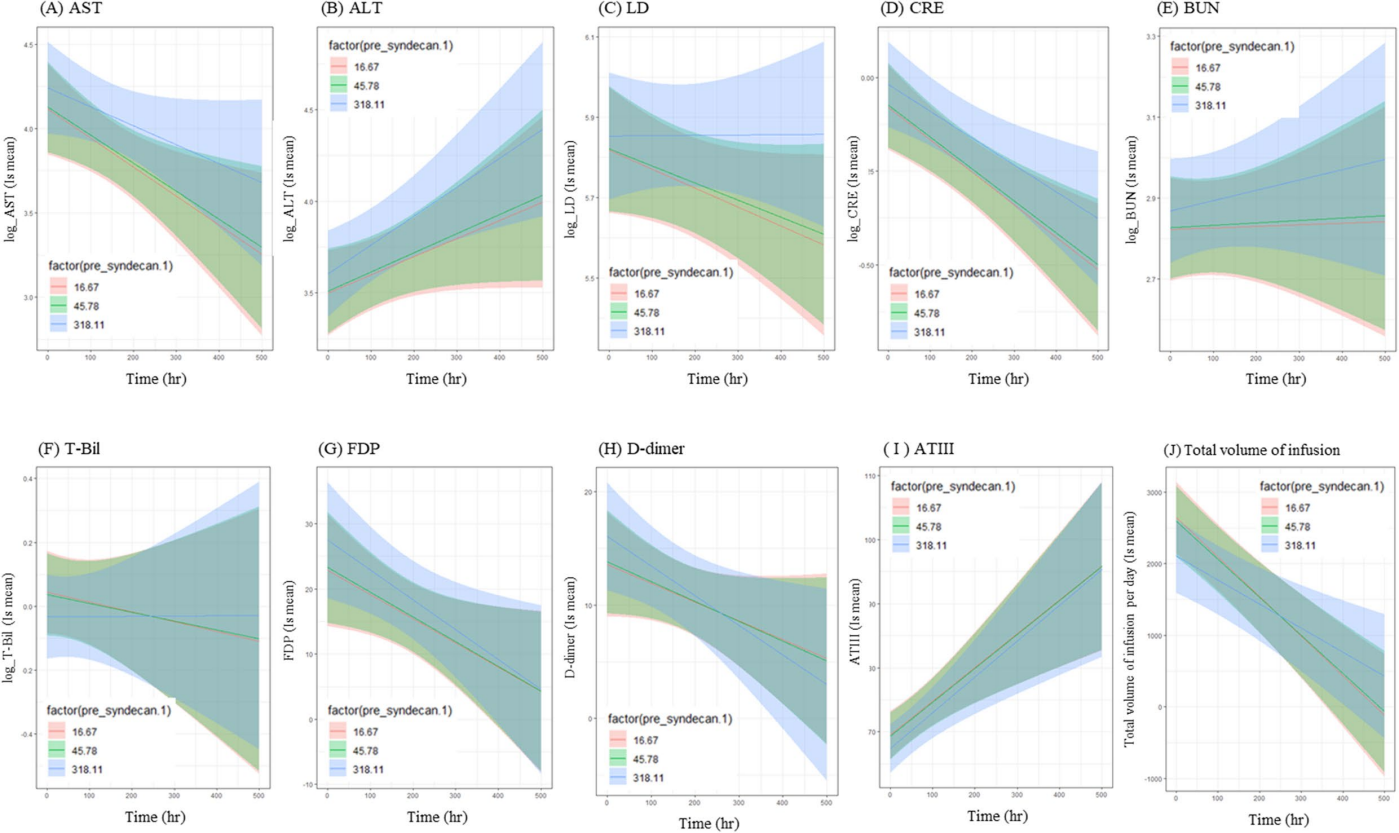
Predicted value of each parameter over time in patients with serum SDC-1 levels of the day prior of 16.67 ng/mL (red line), 45.78 ng/mL (green line), and 318.11 ng/mL (blue line).

Figure 1 shows scatter plots of actual values of each parameter over time in critically ill patients with serum SDC-1 levels of the prior day divided into 4 groups (< 16.67 ng/mL, 16.67–45.78 ng/mL, 45.78–318.11 ng/mL and ≥ 318.11 ng/mL) based on the 10%, median and 90% level. Figure 2 depicts the linear predicted value of each parameter over time in patients with serum SDC-1 levels of the prior day of 16.67 ng/mL, 45.78 ng/mL and 318.11 ng/mL, which represent the 10th, median and 90th percentile of serum SDC-1, respectively, derived from the mixed effects models summarized in Table 2.

AST, ALT, LD, CRE, BUN, FDP and D-dimer levels were higher in patients with serum SDC-1 levels of 318.11 ng/mL (high serum SDC-1 levels) than in patients with serum SDC-1 levels of 16.67 ng/mL and 45.78 ng/mL (low serum SDC-1 levels). Total volume of infusion per day, T-Bil and AT III levels were lower in patients with high serum SDC-1 levels than in patients with low serum SDC-1 levels.

Differences in AST, ALT, LD, CRE and BUN levels among patients with high and low serum SDC-1 levels increased over time. In contrast, differences in FDP and AT III levels among these patients changed over time but converged to the same value at the end of observational period. Moreover, differences in D-dimer, T-Bill levels and total volume of infusion per day among these patients reversed over time.

### Relationship between serum SDC-1 levels and mortality in critically ill patients

Time-varying Cox proportional hazards regression showed that increasing serum SDC-1 level was significantly correlated with mortality after adjustment for age (hazard ratio for an increase in SDC-1 from 24.23 to 143.91 = 2.08, 95% CI 1.24–3.49, *P* = 0.006; Table 3).

**Table 3:** Time-dependent Cox proportional hazards regression analysis of mortality in critically ill patients.

Factor	HR	95% LCI	95% UCI	*P*-value
Serum SDC-1 level (IQR: 24.2–143.9)	2.08	1.24	3.49	0.006
Age (IQR: 54–78.5)	1.93	0.53	6.95	0.317

*HR* hazard ratio for an increase in each factor from the 25 percentile to the 75 percentile, *IQR* interquartile range, *LCL* lower confidence limit, *UCL* upper confidence limit, *SDC-1* syndecan-1.

## Discussion

Serum SDC-1 levels measured the day before laboratory parameter measurements had significant main effects on several outcomes including AST, ALT, T-Bil, CRE, BUN, FDP and AT III, and showed a tendency towards having an effect on LD and D-dimer. Additionally, serum SDC-1 levels of the prior day significantly modified the effect between time and several outcomes, including AST, ALT, LD, CRE, BUN and T-Bil. However, it is important to note that the results of the interactions shown in Table 2 alone only suggest that these factors change the relationship between measurement time and SDC-1. It is therefore important to carefully determine the relationship based on Fig. 2 and clinical observations. Moreover, increasing serum SDC-1 level was a significant risk factor for mortality.

Several previous reports have suggested an association of endothelial glycocalyx injury monitored using circulating biomarkers such as SDC-1^21,24^, heparin sulfate^26,27^ and hyaluronan^26^, and mortality in patients with sepsis, severe sepsis or septic shock. Consistent with previous reports, the present data using serum SDC-1 showed an association between endothelial injury and mortality in critically ill patients. These results support the theory that endothelial injury occurs in the process of organ dysfunction, in which endothelial injury leads to microcirculatory dysfunction with subsequent organ ischemia and organ damage^28,29^.

AST, ALT, LD, CRE and BUN levels were higher in patients with high serum SDC-1 levels of the prior day than in those with low levels, with differences increasing with time from ICU admission. Fold-change in AST, ALT, LD, CRE and BUN for a change in SDC-1 from the median to the 90th percentile was 12%, 10%, 3%, 6% and 4%, respectively (Table S1). Oda et al. performed a retrospective observational study showing that AST and BUN were closely related to serum SDC-1 in patients who underwent comprehensive medical examinations^19^. Johansson et al. showed that serum SDC-1 was correlated with CRE in septic patients who received noradrenaline^23^. Given that AST and ALT are elevated in liver injury, and CRE and BUN are increased in renal injury, serum SDC-1 may be a useful biomarker for daily monitoring to detect early signs of liver and kidney injury in critically ill patients. In particular, several studies have reported an association between serum SDC-1 level and acute kidney injury (AKI). Puskarich et al. reported that among patients with severe sepsis or septic shock, serum SDC-1 levels were significantly higher in patients with acute kidney injury (AKI) than in those without^21^. Similarly, Libório et al. showed in a cohort study of an outbreak of leptospirosis among military personnel that patients with leptospirosis-associated AKI had significantly elevated levels of SDC-1, in addition to intercellular adhesion molecule-1, compared to leptospirosis patients with no AKI^30^. However, LD is a cytoplasmic enzyme that is widely expressed in tissues such as the heart, lungs, kidneys liver, and brain, and is released by these tissues when they are damaged^31,32^. Further studies are needed to elucidate the relationship between SDC-1 level and heart, lung and brain injuries in critically ill patients.

In contrast, T-Bil and total volume of infusion per day were lower in patients with high serum SDC-1 levels of the prior day than in those with low levels, and serum SDC-1 was negatively correlated with T-Bil. Fold-change in T-Bil for a change in SDC-1 from the median to the 90th percentile was -7% (Table S1). However, T-Bil levels were within the normal range in almost all patients at ICU admission in the present study, and changes in T-Bil within the normal range may not be reflected in the serum SDC-1 level. Consistent with the present data, a retrospective observational study of patients who underwent comprehensive medical examinations also reported that T-Bil was negatively correlated with serum SDC-1 levels^19^. In contrast, however, Johansson et al. reported that serum SDC-1 was positively correlated with bilirubin levels in septic patients who received noradrenaline^23^. In this study, we also found that patients with high levels of T-Bil showed a tendency towards having high serum SDC-1 levels (data not shown), and it is possible that we may have identified a positive association between T-Bil and SDC-1 if there was a sufficiently large number of patients with high level of T-Bil in our study. However, a number of factors can affect total volume of infusion per day, including those that have nothing to do with the patient. The validity of our results, therefore, needs further verification.

While serum SDC-1 had significant main effects on several outcomes including AT III, FDP and D-dimer, it did not modify the effect between time and these outcomes. Differences in FDP, D-dimer and AT III for a change in SDC-1 from the median to the 90th percentile were 4.19, 2.23 and − 1.93, respectively (Table S2). AT III levels were lower in patients with high serum SDC-1 levels of the prior day than in those with low levels. AT III levels decrease in critically ill patients due to loss of AT III from the circulation into tissues through increased blood vessel permeability induced by endothelial damage, and decreased production of AT III in the liver and inactivation of AT III by the enzyme elastase^33,34^. Thus, considering that serum SDC-1 is the core protein of heparan sulfate proteoglycan, a constituent of endothelial glycocalyx, it makes sense that serum SDC-1 had significant main effects on AT III.

FDP and D-dimer are the most important markers for diagnosis of disseminated intravascular coagulation (DIC), which is induced by endothelial injury^36^. In a prospective observational study of patients with sepsis, Ikeda et al. reported that there was a weak negative correlation between serum SDC-1 levels and AT III^36^. Moreover, serum SDC-1 levels on Day 1 were significantly higher in patients with than without DIC and had strong discriminative power for predicting both DIC development and mortality. These results are mostly consistent with the present data, suggesting that serum SDC-1 may be a useful biomarker for daily monitoring of the development of DIC.

Differences in AT III and FDP between patients with high serum SDC-1 levels of the prior day and those with low levels changed over time but converged to the same value at the end of observational period. Further, differences in D-dimer between patients with high serum SDC-1 levels of the prior day and those with low levels reversed with time. Although the mechanisms underlying these changes are unclear, these parameters may have been affected by medical interventions such as AT III replacement therapy and treatment for DIC.

Taken together, our data suggest that serum SDC-1 may be a useful biomarker for daily monitoring of critically ill patients to detect early signs of kidney, liver and coagulation system dysfunctions. Systemic inflammatory conditions such as sepsis induce multiple organ dysfunctions in critically ill patients. Given the wide distribution of endothelial glycocalyx throughout the body, serum SDC-1 may also be a useful biomarker for daily monitoring of injuries to other organs such as the heart, lungs and brain.

There were several limitations in the present study. First, in addition to endothelial glycocalyx, SDC-1 is also expressed in other organs. We did not evaluate SDC-1 expression in other organs or its associated effects. Second, we were unable to evaluate the extent of endothelium injury and serum cytokine levels (e.g., IL-6, TNF-a, NGAL). Third, the sample size was small and data were obtained from a single institution. Finally, although a straight line about the mean with a narrow confidence interval may provide more salient information, most of the results have a large variance due to the small amount of available data. Thus, further large scale studies are needed to confirm our findings.

In conclusion, serum SDC-1 may be a useful biomarker for daily monitoring to detect early signs of kidney, liver and coagulation system dysfunction, in addition to endothelium injury. Additionally, elevated serum SDC-1 levels may be an important risk factor for mortality in critically ill patients. To verify the usefulness of serum SDC-1 for early detection of these organ dysfunctions, further large-scale studies are warranted.

## Methods

### Patients

This single-center retrospective observational study was conducted at Gifu University Hospital, which is affiliated with Gifu University (Gifu, Japan). Patients admitted to the intensive care unit (ICU) at Gifu University Hospital from March 2019 to February 2020 were enrolled in the present study. Patients who were younger than 18 years old, were undergoing hemodiafiltration, and were discharged from the ICU within 72 h were excluded from the analysis.

### Ethics approval and consent to participate

The investigation conformed with the principles outlined in the Declaration of Helsinki^37^. Ethics approval was obtained from the medical ethics committee of Gifu University Graduate School of Medicine, Gifu, Japan (Institutional review board approval No. 2018-167). All patients provided written informed consent before all study-related procedures. Before initiation, the study was registered to the UMIN Clinical Trials Registry (registry number: UMIN000036261).

### Data collection and study design

On admission to the ICU, blood was routinely sampled every morning from eligible patients, and data from blood samples obtained while patients were in the ICU were used in the present analysis. All laboratory data except serum SDC-1, volume of infusion of maintenance fluid and extracellular fluid, and other patient demographics were extracted from the hospital’s electronic medical records. Serum SDC-1 concentrations were measured using an enzyme-linked immunosorbent assay (950.640.192; Diaclone, Besancon, Cedex, France). These data were retrospectively analyzed.

### Statistical analysis

Patients’ baseline characteristics are presented as median and interquartile range (IQR) for continuous variables, and frequency and proportion for categorical variables. The aim of the study was to identify organ dysfunction that may be amenable to early detection through daily monitoring of SDC-1 levels. To evaluate the effect of SDC-1 on laboratory parameters measured the day after SDC-1 measurement with consideration for repeated measures, we constructed linear mixed effects models, with each parameter as an outcome variable. The models were adjusted for age and sex, and included the interaction term between measurement time and SDC-1. If the interaction term was statistically significant, SDC-1 was determined to have a modifying effect. Additionally, we confirmed the effect of SDC-1 using the main effect of SDC-1, regardless of whether or not the interaction was statistically significant. To normalize residual values, creatine kinase (CK), aspartate aminotransferase (AST), alanine aminotransferase (ALT), lactate dehydrogenase (LD), alkaline phosphatase (ALP), cholinesterase (ChE), creatinine (CRE), blood urea nitrogen (BUN), triglycerides (TG) and T-bilirubin (T-Bil) were natural logarithmically transformed. The *q*-value was calculated using the Benjamini–Hochberg method to determine the false discovery rate (FDR)^38^. A *q*-value less than 0.05 indicates a significant FDR. A time-dependent Cox proportional hazards regression model with adjustment for age was used to evaluate the effect of serum SDC-1 level on mortality. Serum SDC-1 levels were treated as a time-varying variable. A two-sided significance level of 5% was used to indicate statistical significance. R version 3.6.2 was used for all analyses (www.r-project.org).

## Supplementary Information

Supplementary Information
