# Supplementary material for: Serum syndecan-1 reflects organ dysfunction in critically ill patients
Source: Sci Rep. 2021 Apr 23;11:8864. doi: 10.1038/s41598-021-88303-7 (PMC8065146; doi:10.1038/s41598-021-88303-7)
Supplement: Supplementary file 1 — Supplementary Information [file 41598_2021_88303_MOESM1_ESM.docx]

**Table S1.** Fold-change in each parameter for a change in syndecan-1 from the median to the 90th percentile.

| Variable | Fold-change  (exp(coefficient)) | 95% LCI | 95% UCI |
| --- | --- | --- | --- |
| AST | 1.12 | 1.05 | 1.20 |
| ALT | 1.10 | 1.03 | 1.17 |
| LD | 1.03 | 1.00 | 1.07 |
| CRE | 1.06 | 1.03 | 1.08 |
| BUN | 1.04 | 1.01 | 1.08 |
| T-Bil | 0.93 | 0.90 | 0.97 |

AST: aspartate aminotransferase; ALT: alanine aminotransferase; LD: lactate dehydrogenase, CRE: creatinine; BUN: blood urea nitrogen; T-Bil: total-bilirubin; LCL: lower confidence limit, UCL: upper confidence limit.

**Table S2.** Difference in each parameter for a change in syndecan-1 from the median to the 90th percentile

| Variable | Difference | 95% LCI | 95% UCI |
| --- | --- | --- | --- |
| FDP | 4.19 | 0.63 | 7.76 |
| D-dimer | 2.23 | 0.37 | 4.10 |
| ATIII | -1.93 | -3.56 | -0.29 |

FDP: fibrin degradation product; AT III: antithrombin III, LCL: lower confidence limit, UCL: upper confidence limit.
